# Triptolide Suppresses NF-κB-Mediated Inflammatory Responses and Activates Expression of Nrf2-Mediated Antioxidant Genes to Alleviate Caerulein-Induced Acute Pancreatitis

**DOI:** 10.3390/ijms23031252

**Published:** 2022-01-23

**Authors:** Jing Yang, Xujiao Tang, Xue Ke, Yutong Dai, Jinsong Shi

**Affiliations:** School of Pharmaceutical Sciences, Jiangnan University, Wuxi 214122, China; 6201507011@stu.jiangnan.edu.cn (X.T.); kexue0108@163.com (X.K.); yutongdai29@163.com (Y.D.)

**Keywords:** acute pancreatitis, triptolide, inflammatory responses, oxidative stress

## Abstract

Triptolide (TP), the main active ingredient of *Tripterygium wilfordii Hook.f.*, displays potent anti-inflammatory, antioxidant, and antiproliferative activities. In the present study, the effect of TP on acute pancreatitis and the underlying mechanisms of the disease were investigated using a caerulein-induced animal model of acute pancreatitis (AP) and an in vitro cell model. In vivo, pretreatment with TP notably ameliorated pancreatic damage, shown as the improvement in serum amylase and lipase levels and pancreatic morphology. Meanwhile, TP modulated the infiltration of neutrophils and macrophages (Ly6G staining and CD68 staining) and decreased the levels of proinflammatory factors (TNF-α and IL-6) through inhibiting the transactivation of nuclear factor-κB (NF-κB) in caerulein-treated mice. Furthermore, TP reverted changes in oxidative stress markers, including pancreatic glutathione (GSH), superoxide dismutase (SOD), and malondialdehyde (MDA), in acute pancreatitis mice. Additionally, TP pretreatment inhibited intracellular reactive oxygen species (ROS) levels via upregulated nuclear factor erythroid 2-related factor 2 (Nrf2) expression and Nrf2-regulated redox genes expression (HO-1, SOD1, GPx1 and NQO1) in vitro. Taken together, our data suggest that TP exert protection against pancreatic inflammation and tissue damage by inhibiting NF-κB transactivation, modulating immune cell responses and activating the Nrf2-mediated antioxidative system, thereby alleviating acute pancreatitis.

## 1. Introduction

Acute pancreatitis (AP) is a common systemic inflammatory process originating from the pancreas. Most cases of AP are mild and self-limited, but approximately 15–20% of patients develop severe acute pancreatitis. Although diagnosis and treatment technology have been greatly improved in recent years, the overall mortality of the disease has not improved significantly, affecting up to 20% of patients with severe acute pancreatitis [1,2]. Thus, a challenge still lies ahead in the prevention of acute pancreatitis.

Increasingly, evidence has implied that inflammation and oxidative stress are the dominant factors in the process of AP [3,4]. Irrespective of the causative factor, the damage originates in the pancreatic acinar cells, triggering uncontrolled immune cell infiltration and excessive inflammatory cytokine secretion in the pancreas. These events ultimately lead to an inflammatory response and oxidative stress, thereby further aggravating the tissue damage and edema. Nuclear factor-kappa B (NF-κB) is the central driving factor of the inflammatory response which regulates the expression of numerous inflammatory cytokines (such as TNF-α, IL-6, and IL-1β) and is closely related to AP severity in mice [5]. In addition, the progression of AP can also increase the production of ROS and decrease the expression of SOD, an enzyme that removes superoxide anions, therefore inducing excessive oxidative stress [6]. Both the activities and expression of these antioxidant enzymes are regulated by the Nrf2 signaling pathway. Recent studies have underlined the fact that Nrf2 adjusts oxidative stress and the inflammatory response by regulating the expression of genes coding for antioxidant, anti-inflammatory and detoxifying proteins, which further play a significant role in the pathophysiology of various inflammatory diseases, including AP and hepatitis [7]. Consequently, a potential strategy to ameliorate AP is focused on the suppression of oxidative stress and/or inflammation

As a diterpene triepoxide originally purified from *Tripterygium wilfordii Hook.f.* (TWHF), triptolide (TP) displays a variety of bioactivities, such as anti-inflammatory, immunomodulatory, antioxidant, and anti-proliferation activities, and has attracted tremendous scholarly interest [8,9]. Due to its narrow therapeutic window and the high rate of side effects, the wide application of TP is limited [10,11]. Recently, in lipopolysaccharide (LPS)-induced liver injury, TP was shown to regulate the Nrf2 and NF-κB signaling pathways and alleviate oxidative stress and inflammation, which offers a novel insight for the application of TP in inflammatory diseases [12,13].

However, currently, few studies have reported the pharmacological effects of TP during AP treatment. The present study evaluated the potential beneficial effect of TP on AP and explored the underlying cellular mechanism. These results provide a theoretical basis for the rational application of TP and offer new treatment targets and effective treatment measures for acute pancreatitis.

## 2. Results

### 2.1. Triptolide Ameliorated Pancreatic Damage in Caerulein-Induced Acute Pancreatitis Mice

**Figure 1:**
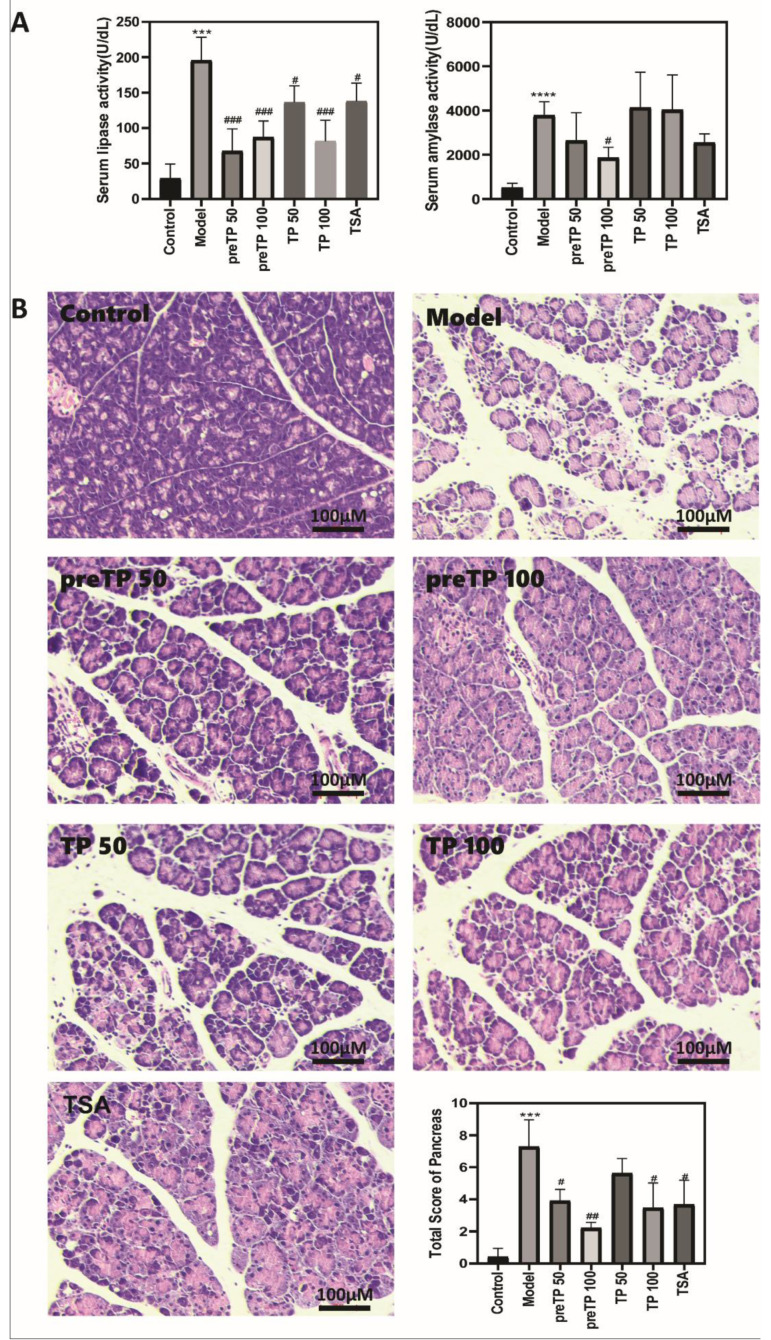
Triptolide-ameliorated pancreatic damage in mice with caerulein-induced acute pancreatitis. (**A**) Serum lipase and serum amylase levels; (**B**) hematoxylin and eosin stained (H&E) section of pancreas. Data shown are means ± SEM. *** *p* < 0.001, and **** *p* < 0.0001 compared with control group. ^#^ *p* < 0.05, ^##^ *p* < 0.01, and ^###^ *p* < 0.001 compared with model group. Triptolide is abbreviated as TP, and tanshinone IIA is abbreviated as TSA.

The combination of caerulein and LPS was used to induce the AP model in ICR mice with the advantages of non-invasiveness, easy induction and reproducibility. As shown in Figure 1A, the serum lipase and α-amylase levels increased dramatically, indicating that pancreatic injury happened. Serum lipase levels were notably attenuated by both pretreatment and therapeutic treatment with TP, while serum α-amylase levels were notably attenuated by pretreatment with TP alone, which confirms that TP alleviates pancreatic damage of AP in mice. H&E staining of pancreatic sections further indicated that caerulein-induced AP showed marked isolation of pancreatic lobes and acinar cells and patchy parenchymal necrosis, together with neutrophil infiltration of the ductal area, interstitial space and parenchyma. TP protected against caerulein-induced pancreatic damage, including edema, inflammation and necrosis; pretreatment with 100 µg/kg TP was especially effective (Figure 1B).

### 2.2. Triptolide Decreases Inflammatory Cell Infiltration and Cytokine Production in Caerulein-Induced Acute Pancreatitis Model

**Figure 2:**
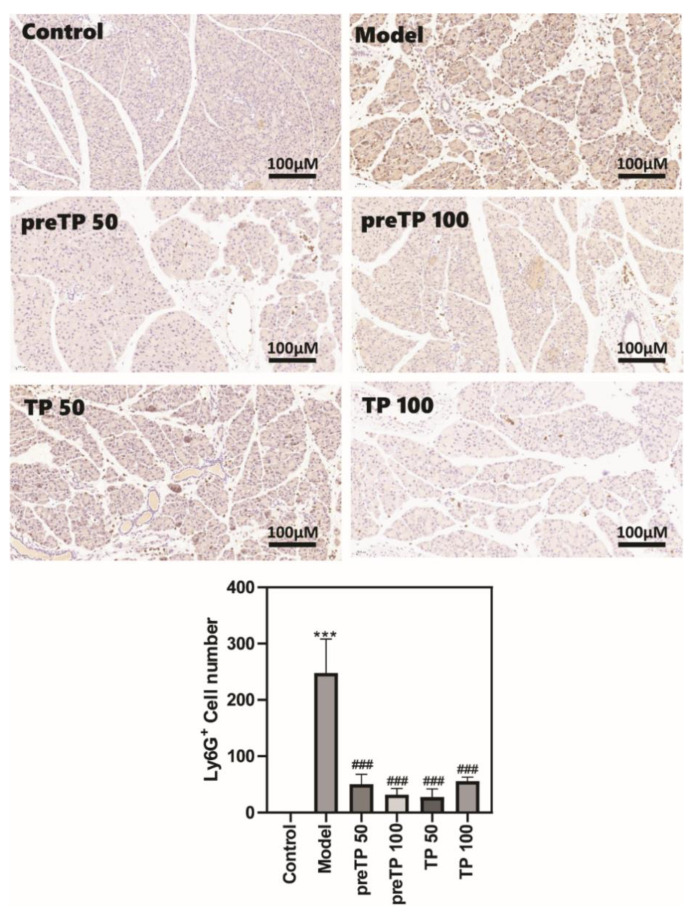
Triptolide decreased neutrophil infiltration in the mice with caerulein-induced acute pancreatitis. Data shown are means ± SEM. *** *p* < 0.001 compared with control group. ^###^ *p* < 0.001 compared with the model group.

**Figure 3:**
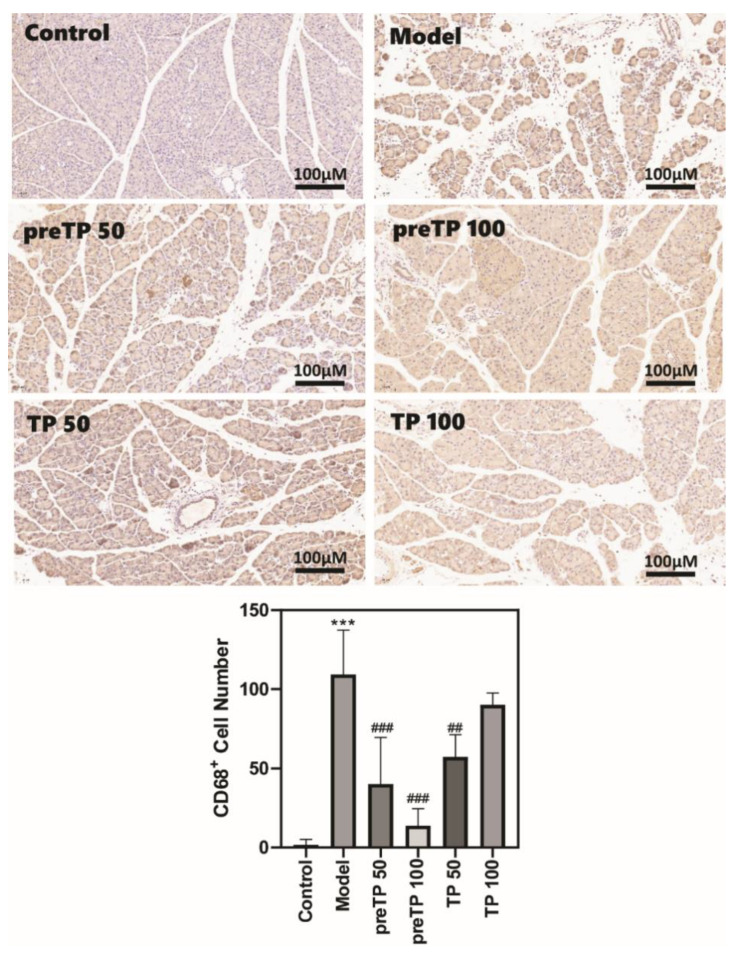
Triptolide decreased macrophage infiltration in mice with caerulein-induced acute pancreatitis. Data shown are means ± SEM. *** *p* < 0.001 compared with control group. ^##^ *p* < 0.01, and ^###^ *p* < 0.001 compared with the model group.

**Figure 4:**
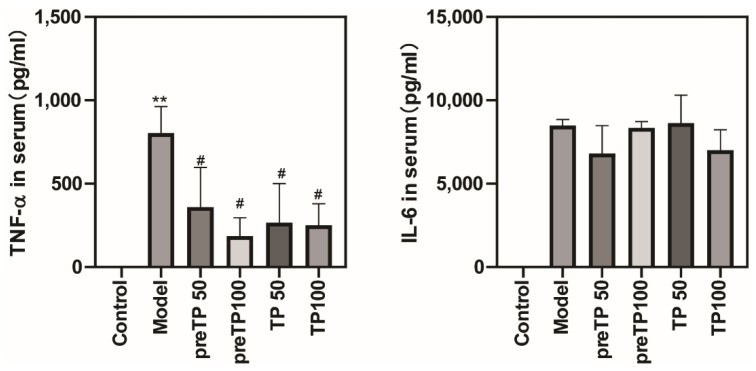
Triptolide reduced inflammatory factors in mice with caerulein-induced acute pancreatitis. Serum TNF-α and IL-6 were measured by ELISA. Data shown are means ± SEM. ** *p* < 0.01 compared with control group. ^#^ *p* < 0.05 compared with the model group. Triptolide is represented by TP, and tanshinone IIA is represented by TSA.

Abundant inflammatory cell infiltration, including of neutrophils and macrophages, has a prejudicial impact on the development of diseases. We investigated the accumulations of neutrophils and macrophages in the pancreas using immunohistochemistry. As shown in Figure 2 and Figure 3, Ly6G+ neutrophils and CD68+ macrophages were abundantly recruited in the model group compared with the normal control group. Then, we further analysed inflammatory cytokine production in serum using ELISA. The levels of inflammatory cytokines (TNF-α and IL-6) were also significantly elevated in the model group compared with the normal control group (Figure 4). The TP treatment groups presented fewer neutrophils and macrophages in the pancreas. Consistently, the serum TNF-α level was significantly decreased with TP treatment, and the serum IL-6 level was slightly reduced in the TP treatment group.

### 2.3. Triptolide Inhibits NF-κB Activation in Caerulein-Induced Acute Pancreatitis Model

**Figure 5:**
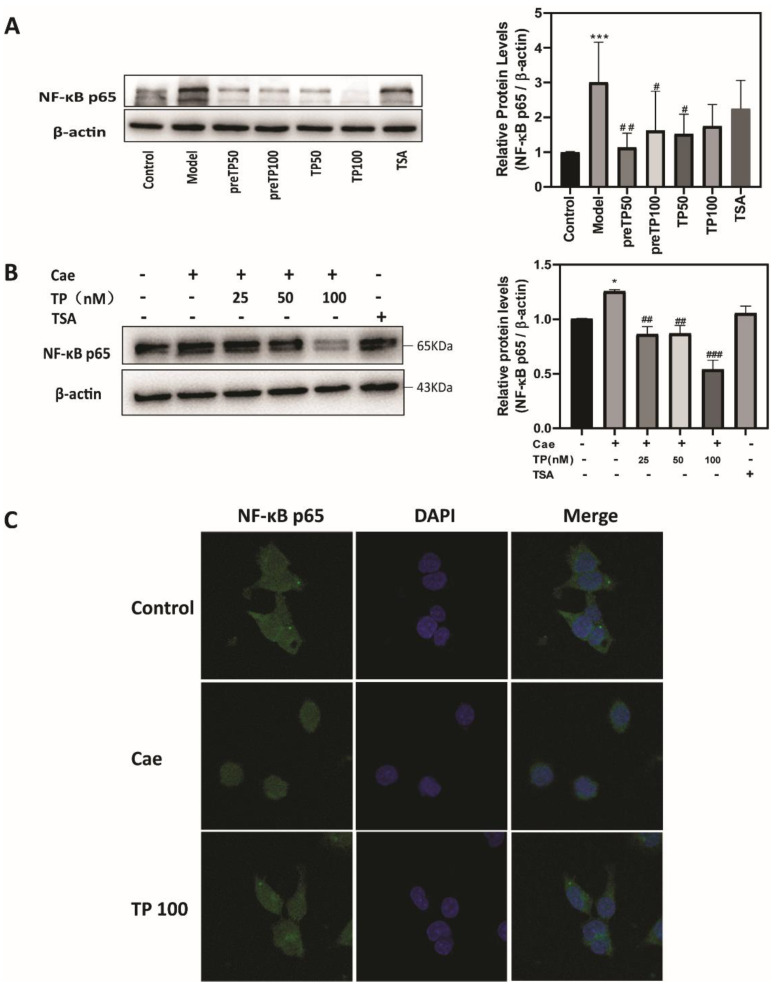
Triptolide inhibited NF-κB activation in a caerulein-induced acute pancreatitis model. (**A**) The expression of pancreatic NF-κB in mice, (**B**) the expression of NF-κB in 266-6 cells, and (**C**) immunofluorescence staining of NF-κB in 266-6 cells. Data shown are means ± SEM. * *p* < 0.05, *** *p* < 0.001 compared with control group. ^#^ *p* < 0.05, ^##^ *p* < 0.01, and ^###^ *p* < 0.001 compared with model group. Triptolide is represented by TP, and tanshinone IIA is represented by TSA.

NF-κB is activated in early-phase acute pancreatitis and regulates the expression of inflammatory factors [14]. Therefore, we evaluated the expression of NF-κB p65 in an acute pancreatitis model via Western blot analysis. Figure 5A shows that the administration of TP markedly reduced the activation of NF-κB compared with that in the model group in vivo. Consistent with the in vivo results, TP pretreatment remarkably inhibited the NF-κB p65 up-regulation induced by caerulein in 266-6 cells, especially at 100 nM (Figure 5B). In addition, TP pretreatment significantly inhibited the nuclear translocation of NF-κB p65 (Figure 5C). Altogether, these results further confirm that TP inhibited inflammatory development and alleviated the pancreatic injury.

### 2.4. Triptolide Alleviates Oxidative Stress via Nrf2 in a Caerulein-Induced Acute Pancreatitis Model

**Figure 6:**
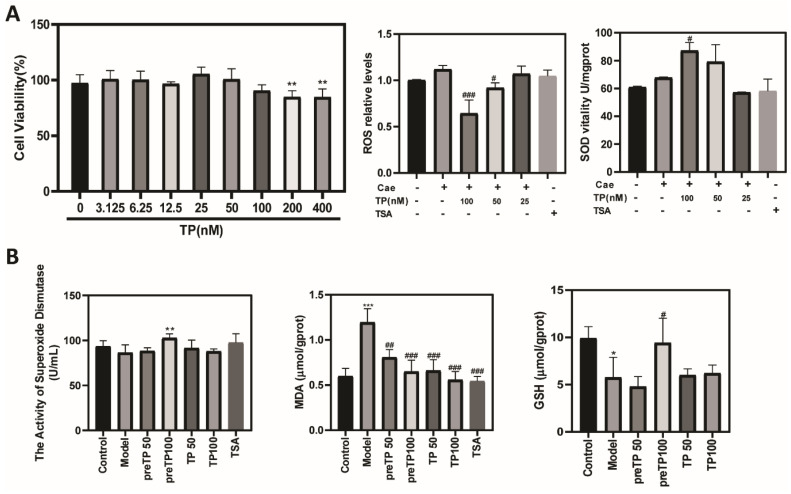
Triptolide alleviates oxidative stress in a caerulein-induced acute pancreatitis model. (**A**) The levels of ROS and SOD in 266-6 cells, and (**B**) the levels of SOD, GSH and MDA in pancreatic tissue. Data shown are means ± SEM. * *p* < 0.05, ** *p* < 0.01, and *** *p* < 0.001 compared with control group. ^#^ *p* < 0.05, ^##^ *p* < 0.01, and ^###^ *p* < 0.001 compared with model group. Triptolide is represented by TP, and tanshinone IIA is represented by TSA.

**Figure 7:**
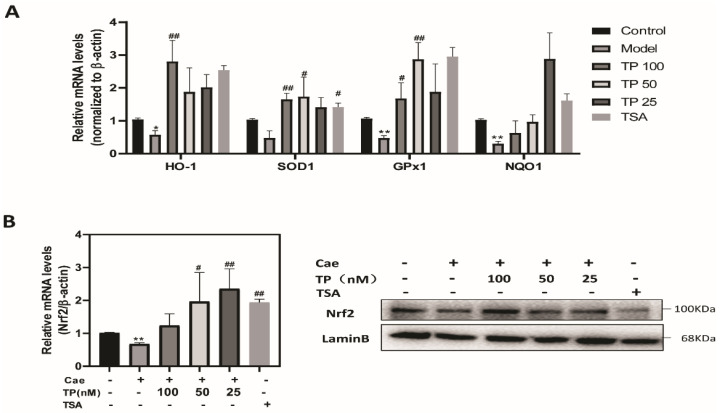
Triptolide activated the Nrf2 signaling pathway in a caerulein-induced acute pancreatitis model. (**A**) The mRNA expression of HO-1, SOD1, GPx1 and NQO1 in 266-6 cells. (**B**) The mRNA and protein expression of Nrf2 in 266-6 cells. Data shown are means ± SEM. * *p* < 0.05, ** *p* < 0.01 compared with control group. ^#^ *p* < 0.05, and ^##^ *p* < 0.01 compared with the model group. Triptolide is represented by TP, and tanshinone IIA is represented by TSA.

Reactive oxygen species (ROS) and damage play an important role in a wide variety of inflammatory diseases, including AP. To further examine the mechanism of how TP exerts its effect, we examined whether TP pretreatment could alleviate oxidative stress in caerulein-induced AP. First, we evaluated the cytotoxic effect of TP on the pancreatic acinar cancer cell line, 266-6. The TP dose we used in subsequent experiments (25, 50 and 100 nM at 12 h) did not cause cytotoxic effects. As shown in Figure 6A, caerulein treatment increased intracellular ROS levels in 266-6 cells compared with the control group, which were decreased significantly by TP pretreatment. Meanwhile, intracellular SOD levels were significantly increased in the TP groups compared with the model group. In agreement with the in vitro results, pancreatic SOD and GSH levels were significantly decreased in the model group, while TP pretreatment dramatically reversed SOD and GSH levels (Figure 6B). As shown in Figure 6B, TP pretreatment effectively revised the up-regulation of the MDA level induced by caerulein in AP mice. Furthermore, we detected the mRNA expression of antioxidant enzymes, including HO-1, SOD1, GPx1 and NQO1. Pretreatment with TP significantly increased the expression of antioxidant enzymes compared with the model group (Figure 7A). Nrf2 is a redox-sensitive transcription factor that becomes activated and translocated into the nucleus in response to oxidative stress [7]. Figure 7B shows that treatment with TP significantly increased the expression and activation of Nrf2 compared with the model group in vitro. Therefore, TP could protect against oxidative stress via activation of the Nrf2 signaling pathway in acute pancreatitis.

## 3. Discussion

In the present study, we identified that TP protected against caerulein-induced acute pancreatitis by attenuating the accumulation of neutrophils and macrophages and reducing the levels of inflammatory cytokines by inhibiting NF-κB inflammatory pathways. In addition, TP upregulated antioxidant enzymes including HO-1, SOD1, and GPx1 NQO1 through Nrf2 activation, further alleviating pancreatic damage.

In acute pancreatitis, irrespective of the causative factor, the primary damage of acinar cells releases various DAMPs that activate local inflammatory responses. Uncontrolled local inflammatory responses further aggravate systemic inflammatory response syndrome (SIRS) and multi-organ dysfunction (MODS) [14]. Thus, focusing on suppressing inflammatory responses may be a promising strategy for the treatment of acute pancreatitis [15]. TP is the main active ingredient derived from the Chinese herb *Tripterygium wilfordii Hook.f.*, which has been used for centuries to treat inflammatory and autoimmune diseases such as rheumatoid arthritis in the clinic. TP has been shown to have potent anti-inflammatory, antioxidant, and antiproliferative activities [16]. Preclinical studies have revealed that TP was effective against rheumatoid arthritis, bone marrow transplantation and cancer in animal models [17,18,19,20]. TP and its derivatives (PG490-88 and F60008) have entered human clinical trials [21,22,23,24]. However, its severe toxicity and poor water solubility restricts the further application of TP in the clinic. It was reported that the IC50 values of TP on all cancer cell lines are in the low nanomole range (average IC50 = 12 nM) at 72 h [25,26,27]. In the present study, we first evaluated the cytotoxic effect of TP on the pancreatic acinar cancer cells, and the dose we used in cells (25, 50 and 100 nM at 12 h) did not cause cytotoxic effects. A study demonstrated that the LD50 for TP administered intravenously was 0.83 mg/kg in mice [28]. In our prior research, after a single intraperitoneal injection of 100 μg/kg TP, there were no changes in different organs (liver, kidney, spleen, thymus, pancreas) in mice in vivo.

In the early stage of acute pancreatitis, once pancreatic injury happens, in response to various inflammatory factors, neutrophils infiltrate the site of injury within minutes and reach a peak within hours, which can not only release cytotoxic signals, but also recruit additional neutrophils and monocytes to aggravate pancreatic damage [29]. In this study, TP pretreatment decreased the number of pancreatic neutrophils and monocytes/macrophages in mice following caerulein-induced injury. At the molecular level, TP was shown to interfere with a number of transcription factors, including NF-κB, p53, NF-AT and HSF-1 [30,31,32,33]. Consistent with previous studies, TP directly suppresses the transactivation of NF-κB in the AP model. More recently, it was shown that TP inhibits the activity of XPB and TFIIH and blocks RNAPII-mediated transcription initiation; hence, it blocks transactivation by all these transcription factors, which may also be an underlying cause of its toxicity [34,35].

In vitro, AP model cells showed increased intracellular ROS levels induced by caerulein, which were decreased significantly by TP pretreatment. In vivo, AP mice showed a remarkable increase in pancreatic MDA content and a decrease in pancreatic SOD and GSH levels, further verifying a low pancreatic antioxidant capacity. However, TP pretreatment dramatically reversed the changes in parameters of pancreatic oxidative damage. Furthermore, TP pretreatment transactivated the Nrf2 signaling pathway and promoted the transcription of target genes including HO-1, SOD1, GPx1, and NQO1 in pancreatic acinar cells following caerulein-induced injury. These results are consistent with previous reports on lipopolysaccharide-induced liver injury [12]. However, at a high concentration or after a long course, TP could induce oxidative stress and damage in HepG2 cells [36].

In conclusion, we found that TP attenuated the inflammatory response by inhibiting inflammatory cell infiltration through the NF-κB pathway and ameliorated pancreatic damage by improving antioxidant activities through the Nrf2 pathway during AP (Figure 5). Our findings suggest the therapeutic potential of TP as a natural drug for treating acute pancreatitis.

## 4. Material and Methods

### 4.1. Chemicals and Reagents

Triptolide (purity >98%) was purchased from Bide Pharmatech Ltd. (Shanghai, China). Caerulein was purchased from Nanjing Peptide (Nanjing, China). LPS was purchased from Aladdin (Shanghai, China). Antibodies against β-actin were obtained from Beyotime Biotechnology (Shanghai, China). Antibodies against NF-κB(p65) and Nrf2 were obtained from Cell Signaling Technology, Inc. (Beverly, MA, USA).

### 4.2. Animal Model of Acute Pancreatitis and Treatment

All animal experiments were approved by the Animal Research Committee of Jiangnan University (JN.No20201130i0240131[348]). Male ICR mice (20 ± 2 g) were purchased from Cavens (Changzhou, Jiangsu, China). Mice were divided randomly into experimental groups (*n* = 6) as follows: (1) Control, (2) AP Model, (3) TP preventive administration (50 μg/kg, preTP50), (4) TP preventive administration (100 μg/kg, preTP100), (5) TP therapy group (50 μg/kg, TP50), (6) TP therapy group (100 μg/kg, TP100) and (7) tanshinone IIA therapy group (25 mg/kg, TSA). The acute pancreatitis model was induced by hourly intraperitoneal injection of caerulein (200 µg/kg) for 10 h and intraperitoneal injection of LPS (5 mg/kg) at 1 h after the last caerulein injection. TP was dissolved by 0.5% carboxymethylcellulose sodium (CMC-Na) suspension. The preventive administration groups were pretreated with TP 0.5 h in advance by gavage. The treatment groups were intragastrically administrated TP one hour after the first caerulein injection. Then, serum and pancreas tissues were harvested for subsequent assays. Twelve hours after the first caerulein injection, mice were euthanized with phenobarbital sodium by intraperitoneal injection, and serum and pancreatic tissues were harvested for subsequent assays.

### 4.3. Cell Culture and Treatment

Next, 266-6 cells were purchased from Cobioer Biosciences Co, Ltd. (Nanjing, China) and were cultured in DMEM with 10% FBS and 1% Penicillin-Streptomycin solution. Cell cultures were then maintained in a humidified atmosphere at 37 °C with 5% CO_2_. The cells were pretreated with TP (25–100 nM, 0.5 h) before stimulation with caerulein (10 nM) for 12 h. Supernatants and cells were then collected for subsequent assays.

### 4.4. Measurement of Serum Lipase and α-Amylase Activity

Blood from mice was collected and centrifuged (4000 rpm, 10 min, 4 °C) to obtain the supernatant for further detection. Serum activity of α-amylase and lipase were detected using assay kits (Jiancheng Biotech, Nanjing, China). All kits were used according to the manufacturer’s instructions.

### 4.5. HE Staining

Fresh pancreas samples were fixed in 4% paraformaldehyde, embedded in paraffin for hematoxylin and eosin staining (H&E) and examined by light microscopy (400×). Two investigators who were blinded to the experimental treatment scored the degree of tissue injury; the scoring standards were described previously [37].

### 4.6. Determination of ROS

Cells were incubated with the fluorescence dye DCFH-DA (Beyotime, Nantong, China) in the dark at 37 °C for 20 min, washed with PBS 3 times, and detected at an excitation wavelength of 488 nm and an emission wavelength of 525 nm.

### 4.7. Determination of SOD, GSH and MDA

The total SOD, GSH and MD activity in 266-6 cells and pancreatic tissues were analysed using the SOD kit, the GSH kit and the MDA kit (Jiancheng Biotech, Nanjing, China), respectively.

### 4.8. Determination of Serum TNF-α and IL-6 Levels

The serum levels of TNF-α and IL-6 were measured by enzyme-linked immunosorbent assay (ELISA) kits (Proteintech, Wuhan, China) according to the manufacturer’s protocols.

### 4.9. Western Blot Analysis

Cells or pancreatic tissues were homogenized in RIPA buffer with protease and phosphatase inhibitors. Protein concentrations were evaluated by the BCA Protein Assay Kit (Beyotime, Nantong, China). Equal amounts of protein were separated in 10% SDS-polyacrylamide gel and transferred to polyvinylidene difluoride membranes. After blocking with non-fat milk or BSA for 1 h, the membranes were probed with primary antibodies, followed by the addition of HRP-labelled secondary antibodies. The bands were visualized with the ECL reagents. The band density was quantified using Image J software.

### 4.10. RNA Isolation and Quantitative Real-Time PCR

Total RNA was obtained with TRIzol reagent (Invitrogen, CA, USA), and cDNAs were synthesized by a reverse transcription reagent kit (Vazyme Biotech, Nanjing, China). Quantitative reverse transcription-PCR was performed using a SYBR Green qPCR Master Mix (Vazyme Biotech, Nanjing, China). Primer sequences are given in Table 1. The relative mRNA expression levels were evaluated by the 2^−ΔΔCT^ method, using β-actin as a reference gene.

### 4.11. Statistical Analysis

Data are expressed as the mean ± SEM. Differences among multiple groups were assessed using one-way analysis of variance followed by Bonferroni’s multiple comparison Test. The results are considered statistically significant at *p* < 0.05. All analyses were conducted using GraphPad Prism 8 software.

## Figures and Tables

**Table 1:** Primer sequences [38].

Target Gene	The Expected Product Size	Forward	Reverse
β-actin	1469 bp	5′-GGCTGTATTCCCCTCCATCG-3′	5′-TGTAGACCATGTAGTGGTCA -3′
HO-1	211 bp	5′-AACAAGCAGAACCCAGTCTATGC-3′	5′-AGGTAGCGGGTATATGCGTGGGCC-3′
SOD1	2348 bp	5′-TGGGTTCCACGTCCATCAGTA-3′	5′-ACCGTCCTTTCCAGCAGTCA-3′
GPx1	341 bp	5′-TCAGTTCGGACACCAGGAGAA-3′	5′-CTCACCATTCACTTCGCACTTC-3′
NQO1	110 bp	5′-CAAGTTTGGCCTCTCTGTGG-3′	5′-AAGCTGCGTCTAACTATATGT-3′
Nrf2	106 bp	5′-TCCGCTGCCATCAGTCAGTC-3′	5′-ATTGTGCCTTCAGCGTGCTTC-3′

## Data Availability

Data available on request from the authors.
